# 3D convective flow in a hybrid nanofluid filled bi-truncated-pyramid equipped with adiabatic cylinders

**DOI:** 10.3389/fchem.2024.1522372

**Published:** 2025-01-22

**Authors:** Mohammed A. Almeshaal

**Affiliations:** Department of Mechanical Engineering, College of Engineering, Imam Mohammad Ibn Saud Islamic University, Riyadh, Saudi Arabia

**Keywords:** 3D natural convection, hybrid nanofluid, bi-truncated-pyramid, regression analysis, FEM

## Abstract

This study consists of an investigation of the 3D convective flow of hybrid nanofluids (HNFs) within a bi-truncated pyramid equipped with adiabatic cylinders, with a focus on the enhancement of natural convection (NC) heat transfer (HT). The use of HNFs, which is based on the combination of two different nanoparticles (NPs), provides improved thermal conductivity and stability, and leads to significant advantages in thermal management systems. Numerical simulations based of the FEM were performed to analyze the effects of Rayleigh number (Ra), nanoparticle volume fraction ( φ ), and cylinders size (D) on the heat transfer and fluid flow (FF) within the pyramid. The results showed that at higher Ra and nanoparticle concentrations a significant enhancement of the HT occurs, and the average Nusselt number (Nu_a_) was increased by up to 23% at a Ra = 10^6^ and φ = 0.045. Concerning the adiabatic cylinders, it was found that the optimal cylinder diameter is D = 0.15, (balance between flow disturbance and heat transfer rate). The outputs of the current study are valuable in the optimization of the hybrid nanofluid applications for advanced thermal management solutions.

## 1 Introduction

With a growing demand for improvements in heat transfer performances, the development of advanced technologies to manage thermal issues more efficiently are one of today’s hottest topics across different engineering and industrial segments. An innovative technique that recently attracted much attention consist of the utilization of HNFs to enhance NC HT. HNFs are formulated via an innovative process in which two different types of NPs are suspended in a base fluid (BF), with the intention to capture substantial heat transfer qualities from each type. For this purpose, a wide variety of applications are found in the literature for natural convection use of hybrid nanofluids. These are used in electronics cooling such as microprocessors or power electronic components, solar thermal systems and building thermal management. Recent works mainly dedicated to the use of HNFs, which is a combination of different nanoparticles, have shown their advantage in the improvement of the convective HT coefficient. Due to their enhanced properties, better stability and optimized thermal performance/cost, the applications of HNFs in convective heat transfer became the interest of several researchers.

Studies related to hybrid nanofluids showed important improvements in thermal conductivity (TC) and HT performance. Omri et al. (2022) presented a study on the performance’s enhancement of a vertical coiled heat exchanger working with CuO-Gp/water HNF. After ultrasonic dispersion (to avoid agglomeration), the authors measured the TC and viscosity of the HNF. It was found that the HT coefficient increased by 23.65% at a 0.2% mass fraction and by up to 79.68% at a 1% mass fraction. In addition, the increase of Reynolds number (Re) caused the augmentation of the HT coefficient. The HT and FF in a wavy cross-flow micro heat exchanger working with HNF have been studied by Ghachem et al. (2021). They considered a 3D configuration and studied the effects of different factors, like the fluid velocity, the waves number, and nanoparticles concentration. The HNF, which combines carbon nanotubes (CNT) and aluminum oxide (Al_2_O_3_) nanoparticles, showed significant improvements in both thermal conductivity and heat transfer rates, especially when the flow velocity was high. The researchers discovered that finding the optimal combinations of these parameters could reduce the size of the heat exchanger while boosting its efficiency. Dero et al. (2022) used the bvp4c solver to study the thermal stability of HNFs in a porous media using. The dual branch framework was used to highlight the effects of viscous dissipation and suction/injection effects on the thermal performances and stability of Cu-Al_2_O_3_/water hybrid nanofluid. Under certain conditions, dual solutions were found, this behavior indicates the complex nature of the thermal and fluid dynamics.

Natural convection corresponds to fluid motions caused by density variations due to temperature gradients. It occurs in several applications such as cooling electronic devices, solar energy, and in buildings. The conventional HT fluids such as water, ethylene glycol, and lubricants frequently lead to low thermal performances. Single nanofluids, are efficient in the enhancements of thermal conductivity and convective heat transfer, but they have limitations related to particle aggregation and sedimentation. The use of hybrid nanofluids solve these problems, in fact dispersing different nanoparticles in a base fluid allows to benefit from the distinct thermal, chemical, and mechanical characteristics of each nanoparticle type to attain better stability, higher TC, and enhanced specific heat capacity. HNFs exhibit enhanced effective thermal conductivity in comparison to conventional nanofluids, leading to enhanced heat transfer efficiency. Jino et al. (2024) introduced a new technique for cooling electric vehicle batteries. They used hybrid nanofluids in inverted right-angled triangular porous cavities. This study emphasises the significance of thermal management in the enhancement of the performances, safety, and longevity of electric vehicle batteries. The authors analyzed the impact of various parameters (Ra, Da, Ha, and φ ), on HT, FF, and irreversibility. The findings demonstrated that the HT rate was considerably increased when an inverted triangular cavity is used compared to traditional square-shaped cavities. Khan et al. (2024) examined the characteristics of a HNF in a permeable quadrantal cavity with internal heat generation. The researchers employed the FEM to solve the nondimensionalized equations. They considered the impact of heat generation on the FF and the HT. The study observed a positive correlation between the φ and Nu_a_, suggesting a notable improvement in heat transport. The NC of MWCNT-Fe_3_O_4_/water HNF was studied by Khan N. H. et al. (2022) with a focus on the impact of changes in Ra, aspect ratio, and φ on the HT and entropy formation. The authors mentioned that Nu_a_ is proportional to Ra but reversely proportional to the aspect ratio. In addition, it was found that the use of MWCNT-Fe_3_O_4_ nanoparticles improves the HT rate. Goudarzi et al. (2020) studied the effects of NP migration caused by the thermophoresis and Brownian motion on the natural convection of Ag-MgO/water HNF in a wavy enclosure. The numerical study considered the diffusion of Ag nanoparticles in MgO-water NF and vice versa. Khan S. A. et al. (2022) investigated numerically the NC of Cu-Al_2_O_3_/water HNF inside an annuli. In addition, they performed a multi-response optimization to identify the optimal parameter to maximize the thermal and hydrodynamic performances. The results indicate that inclination angle and heat flux significantly enhance heat transfer, while the aspect ratio and nanoparticle concentration also play vital roles in improving the thermal performances of the HNF. Çiçek et al. (2023) presented a study on the NC of MWCNT-Fe_3_O_4_ hybrid nanofluid within a differentially heated square enclosure. The study consists of an analysis of the impacts of Ra and nanoparticle volume fraction on thermal and flow behaviors. The authors mentioned that the increase of φ leads to an improvement of Nu_a_ at low Ra values, with a 0.1% volume fraction which is identified as the optimal for the HT enhancement at Ra equal to or greater than 10^4^. Nayak et al. (2022) studied the effect of wavy baffle structures on the HT performance of double-diffusive NC within a C-shaped cavity filled with a HNF. The results indicate that streamlines intensify significantly with increased buoyancy ratio and Rayleigh number, enhancing heat and mass transfer. Furthermore, the presence of wavy baffles markedly improves thermal performance by amplifying local Nusselt numbers, especially at higher Rayleigh numbers. Aich et al. (2023) performed a three-dimensional study on the NC and entropy production in a C-shaped enclosure filled with a CNT–Al_2_O_3_/water HNF. The aim of the study was to examine the effects of the aspect ratio, Ra, and φ on HT, FF, and irreversibility production. It was found that the aspect ratio has a significant effect on the HT and entropy. At high Ra the entropy increases due to the enhancement of the convective HT. In addition, the inclusion of hybrid nanoparticles has a benefic effect on Nu_a_, which is an indication of the improvement of heat transfer performance. Almeshaal et al. (2020) conducted a 3D study on NC within a T-shaped enclosure filled with a water-based CNT–aluminum oxide HNF. The was based on the vorticity-vector potential formalism and it was shown that the increase of the size of the enclosure, Ra, and φ cause and enhancement of the HT rate.

The use of internal obstacles in cavities filled with hybrid nanofluids corresponds to an effective strategy for the enhancement of the convective HT. When strategically placed, the obstacles cause the disruption of the FF, and create secondary vortices that lead to a more uniform temperature distribution and increased heat transfer rates. Hybrid nanofluids, consist of the combination of the superior thermal properties of different nanoparticles, for the enhancement of the thermal conductivity and the convective heat transfer. This method is highly beneficial for applications such as electronic cooling, solar collectors, and energy-efficient building designs. Some recent studies have examined the impact of the internal obstacles, like heat-generating bodies and fins, on the HT performance in cavities filled with HNF. In the study of Hassen et al. (2022) the thermocapillary and buoyancy-driven convection in a cavity containing a heated obstacle filled with a MWCNT-Fe_3_O_4_/oil HNF was numerically studied. The study was based on 2D simulations for the examination of the effects of the obstacle’s position, size, Marangoni number, Ra, and φ on the flow and thermal behavior. The findings indicated that repositioning the obstacle from the bottom to the top of the cavity leads to the optimization of the heat transfer enhancement by over 170%. HT and magnetohydrodynamic (MHD) FF characteristics were studied by Mokaddes Ali et al. (2021) in wavy-walled cavities that held a heat-generating cylinder and a Cu-Al_2_O_3_/water hybrid nanofluid. The study was based of the finite element approach for the investigation of the effects of several parameters, such as the cylinder radius, heater length, hybrid nanoparticle volume fraction, Ra, and Ha, on the flow and thermal behavior. The results showed that high Hartmann number causes the decrease of the fluid motion because of the generated Lorentz force, but high Rayleigh number causes the increase of the flow intensity and the rate of HT. Furthermore, the study shows that at lower cylinder radii, shorter heater lengths, and greater hybrid nanoparticle volume fractions all lead to the enhancement of the rate of HT. Dey and Rathish Kumar (2024) conducted a finite element analysis to investigate the NC in a complex porous enclosure filled with a HNF under thermal non-equilibrium assumption. The study corresponds to an investigation of the effects of Ra, Pr, Da, porosity, and φ , on the HT and FF. The results indicated that the dispersion of the Cu-Al_2_O_3_ hybrid nanoparticles, in the base fluid has a benefic effect on the improvement if the HT and FF in the porous media. The LBM was used by Cheng et al. (2023) to investigate the HNF MHD NC and irreversibilities production. The aim of the research was the examination of the impact of Ha, baffle sizes, and magnetic field inclination on the generation of thermal and frictional entropy in a square cavity. The results indicated that the velocity components and temperature gradient are reduced with the increase of Hartmann number, which results in a reduction in all forms of irreversibility. In contrast, the thermal, frictional, and total entropy are augmented by an increase in the inclination angle. Roy (2022) presented a study on the MHD NC HT and FF of a HNF in a cavity equipped with multiple heat sources. The authors used the finite difference method to solve the dimensionless system of equations. The numerical model was validated by comparing with experimental and numerical results. It was found that Rayleigh number, the number and size of heat sources have a substantial influence on the flow pattern. The NC of a Cu-Al_2_O_3_-water HNF in a square cavity equipped with two heat-generating structures was investigated by Hidki et al. (2023). The authors investigated the impact of Ra, φ , and the presence of fins on the blocks, on the temperature field and flow structures using the finite volume method. The results showed that the maximal temperature reduces with the increase of Ras, which is a result of the improvement of the heat exchange. Furthermore, the temperature decreases by up to 18% when the hybrid nanoparticles are incorporated in the base fluid. It was also indicated that the maximal temperature decreases by up to 12% for Ra ≥ 105 when the fins are installed on the surfaces of the blocks. The NC of a non-isothermal HNF has been computationally analysed by Iftikhar et al. (2023). The flow was influenced by a hot fin and non-linear radiation. The FEM was used for the investigation of the effect of the fin height, nanoparticle volume fraction (Ag and Cu), and non-linear thermal radiation on the HT and entropy production. It was found that the NC flow is improved when the height of the hot fins is increased, which results in a considerable increase of the flow intensity and HT rate of up to 42% and 57%, respectively. Furthermore, the addition of NPs leads to a further enhancement of the heat transfer, while the entropy generation exhibits an inverse trend as the nanoparticle concentration is increased. Islam et al. (2023) studied the magnetohydrodynamic (MHD) NC in a prismatic-shaped enclosure containing Cu-TiO_2_/water HNFs. The authors examined the impact of Ra, Ha, and φ , on HT and FF. It was indicated that an enhancement of the HT performances occurs using hybrid NPs especially at high Ra values, whereas the reverse effect is observed when the magnetic influence is increased. In addition to the numerical study, a sensitivity analysis was conducted using response surface methodology.

Based on the above presented literature review it can be concluded that the studies related to 3D natural convection of hybrid nanofluids in complex geometries are very limited. The 3D convective flow in hybrid nanofluid-filled bi-truncated-pyramid cavities equipped with adiabatic cylinders represents a critical advancement in the application of hybrid nanofluids for the enhancement of the thermal management. The aim of this paper is to investigate the complex interactions of natural convection within such geometrically intricate enclosures, taking in account the role of adiabatic cylinders in disrupting the FF and the creation of secondary vortices that cause the augmentation of the heat transfer. This numerical investigation consists of analyzing the effects of varying Rayleigh numbers, nanoparticle volume fractions, and cylinder size on the thermal and fluid dynamic behaviors within the cavity. This approach highlights the enhancements of the thermal properties of the hybrid nanofluids and provides a detailed understanding of the effects of geometric modifications on the optimization of the heat transfer in practical applications.

## 2 Problem statement and mathematical modeling

**FIGURE 1:**
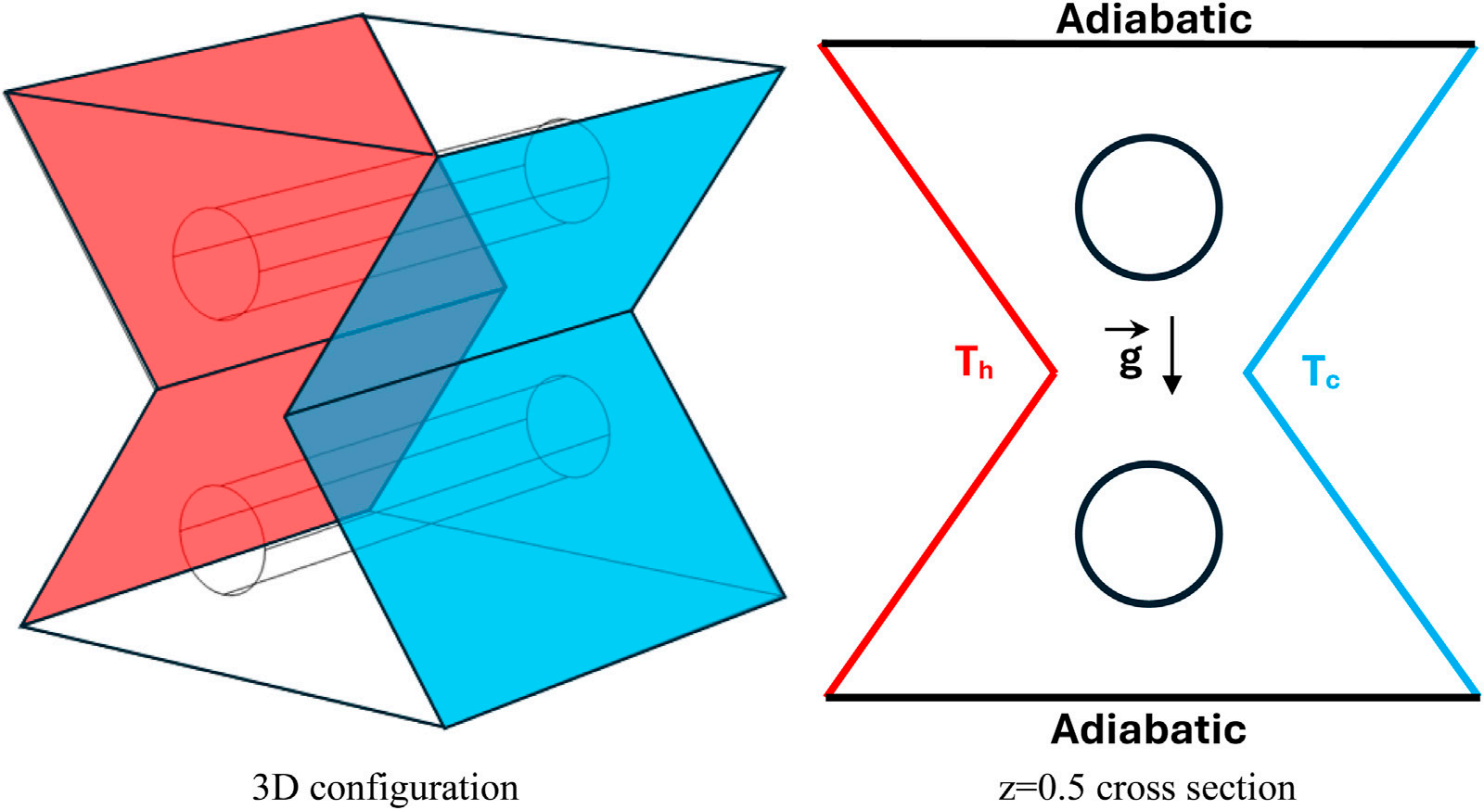
Description of the studied configuration.

The configuration considered in this investigation (Figure 1) consist of a bi-truncated pyramid filled with a hybrid nanofluid and containing two adiabatic cylindrical obstacles (with a diameter of “D”). The left walls are maintained at a higher temperature (T_h_) and the right walls are maintained at a lower temperature (T_c_). The heat transmission across these boundaries is prevented by the inclusion of the adiabatic cylinders (with a diameter of “D”) and the adiabatic pyramid’s top and bottom walls.

The dimensional PDEs Equations 1–3 governing the 3D NC are as follows (Almeshaal et al., 2020):

$$\nabla .\vec{V^{\prime }}=0$$ (1)

$$\frac{\partial \vec{V^{\prime }}}{\partial t^{\prime }}+\left(\vec{V^{\prime }.\;}\vec{\nabla }\right)\vec{V^{\prime }}=-\frac{1}{\rho_{\text{nf}}}\vec{\nabla }P^{\prime }+v∆\vec{V^{\prime }}-\beta_{t}\left(T^{\prime }-{T^{\prime }}_{c}\right)\vec{g}$$ (2)

$$\frac{\partial T^{\prime }}{\partial t^{\prime }}+\vec{V^{\prime }}\;.\nabla T^{\prime }=\alpha_{\text{nf}}∆T^{\prime }$$ (3)

Using the following dimensionless variables:

t=t′L′²α ; x,y,z=x,y,z′L′ ; Vx,Vy,Vz=Vx,Vy,Vz′αL′ ; P=P′ρ αL′ ; and T=T′−Tc′  / Th′−Tc′

The governing equations are converted to the dimensionless form:

$$\nabla .\vec{V}=0$$ (4)

$$\partial \vec{V}/\partial t+\left(\vec{V}.\;\vec{\nabla }\right)\;\vec{V}=-\vec{\nabla }P+\mathrm{Pr}\;\left(V_{\text{nf}}\;/V_{f}\;\right)∆\vec{V}+\left(\beta_{\text{nf}}\;/\beta_{f}\;\right)\text{ Ra }\mathrm{Pr}\;\vec{T_{g}}$$ (5)

$$\partial T/\partial t+\vec{V}.\nabla T=\frac{\alpha_{\text{nf}}}{\alpha_{f}}\nabla ²T$$ (6)

With:

$$\mathrm{Pr}=\frac{\upsilon_{f}}{\alpha_{f}}\;;\text{ and Ra}=\frac{g.\;\beta_{f}\;.∆T\;{.l}^{3}}{\upsilon_{f}.\alpha_{f}}$$

The dimensionless Boundary conditions are:

T=1,at the left walls

T=0,at the right walls

$$\frac{\partial T}{\partial n}=0\text{ at remaining walls}$$

$$V_{x}=V_{y}=V_{z}=0,\text{at all walls}$$

The local and average Nusselt numbers are Equations 7, 8:

$${Nu}_{l}=\left(\frac{k_{\text{nf}}}{k_{f}}\right)\;\frac{\partial T}{\partial n}$$ (7)

And

$${\text{Nu}}_{a}=\int_{0}^{1}\int_{0}^{1}{\text{Nu}}_{l}\;.\text{dy}.\text{dz}$$ (8)

The following expressions Equations 9–15 are used to evaluate the thermophysical properties (Almeshaal et al., 2020; Gal et al., 2022):

Density:

$$\rho_{nf}=\left(1-\varphi \right)\rho_{f}+\varphi \rho_{s}$$ (9)

Heat capacitance:

$${\left(\rho C_{p}\right)}_{nf}=\left(1-\varphi \right){\left(\rho C_{p}\right)}_{f}+\varphi {\left(\rho C_{p}\right)}_{s}$$ (10)

Thermal conductivity.

- For CNT-nanofluid:
$$\frac{k_{nf}}{k_{f}}=\frac{1-\phi +2\varphi \frac{k_{s}}{k_{s}-k_{f}}\ln\left(\frac{k_{s}+k_{f}}{2k_{f}}\right)}{1-\varphi +2\varphi \frac{k_{f}}{k_{s}-k_{f}}\ln\left(\frac{k_{s}+k_{f}}{2k_{f}}\right)}$$ (11)
- For Al2O3-nanofluid:
$$\frac{k_{nf}}{k_{f}}=\frac{k_{s}+2k_{f}-2\varphi \left(k_{f}-k_{s}\right)}{k_{s}+2k_{f}+\varphi \left(k_{f}-k_{s}\right)}$$ (12)

Dynamic viscosity:

- For CNT-nanofluid:
$${µ}_{\mathrm{nf}}={µ}_{f}\left(1+13.5.\varphi +904.4.\varphi^{2}\right)$$ (13)
- For Al2O3-nanofluid:
$$\mu_{nf}=\frac{\mu_{f}}{{\left(1-\varphi \right)}^{2.5}}$$ (14)

The properties of HNF are evaluated as follows:

$$y_{hnf}=y_{CNT}.fr+y_{Al_{2}O_{3}}.\left(1-fr\right)$$ (15)

where y is any thermophysical property and fr is the mass fraction of CNT fixed at 0.15.

The properties of water and NPs are presented in Table 1.

**TABLE 1:** Thermophysical properties of water and NPs.

	Water	CNT	Al_2_O_3_
*Cp [kJ.kg^-1^.K^-1^]*	4.179	0.425	0.765
ρ *[kg.m^-3^]*	997.1	2,600	3,970
*k [W.m^-1^.K^-1^]*	0.613	6,600	40
β *[K^-1^]*	2.1.10^−4^	16.10^−7^	0.85.10^−5^

The FEM, based on the Galerkin’s Weighted Residual technique, is used to solve the set of Equations 4–6 along with the applied boundary conditions.

The Lagrange polynomials are expressed as:

$$H=\sum_{j=1}^{Ns}\delta_{j}^{s}K_{j}$$

where δ is the shape function and K represent the values of the variables at the nodes of the elements. The convergence criterion is fixed at $\left|\frac{f^{n+1}-f^{n}}{f^{n+1}}\right|\leq 10^{-6}$ .

### 2.1 Validation of the numerical model and gris sensitivity analysis

**FIGURE 2:**
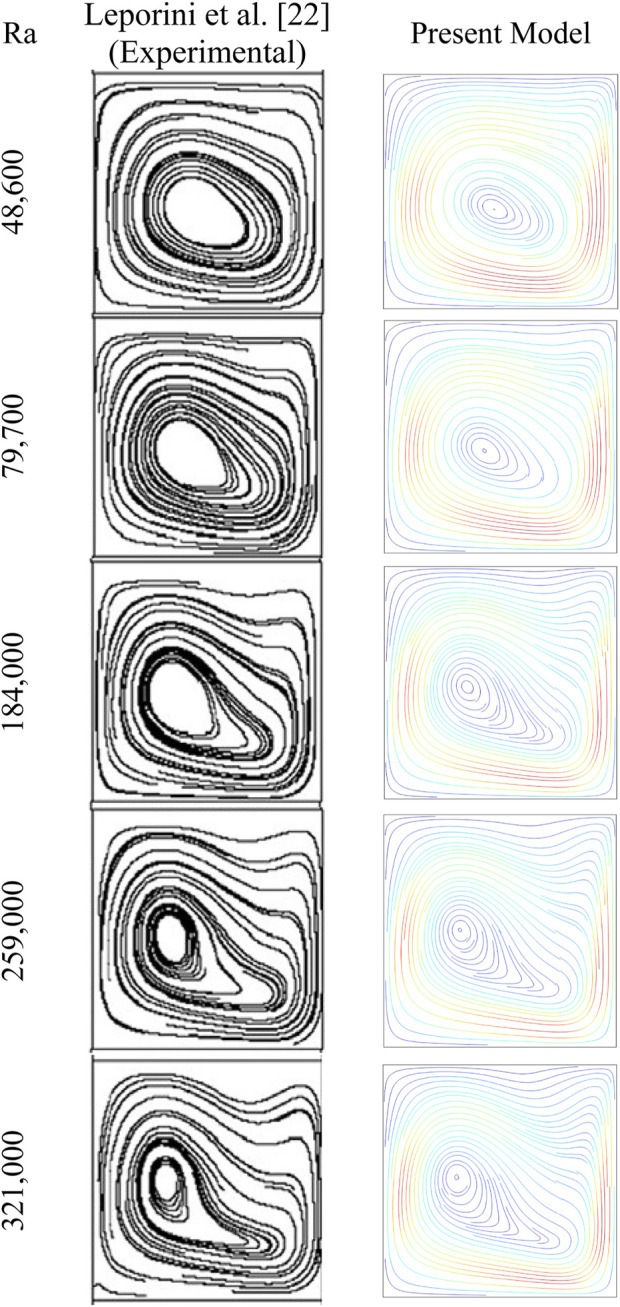
Comparison of the flow structures with the experimental results of Leporini et al. (Leporini et al., 2018), for various Ra Values.

To validate the established numerical model, a qualitative comparison with the experimental results of Leporini et al. (2018) was performed for various Ra values (Figure 2). The work, conducted by Leporini et al. (2018), involved analyzing natural convection in a tilted 3D cavity. The study provided detailed measurements of flow structures and heat transfer rates across a range of Rayleigh numbers. It is clear from the comparison that a good concordance exists between the experimental flow structures provided by Leporini et al. (2018) and those issued from the current numerical model.

The grid sensitivity analysis is illustrated in Table 2, which illustrates the effect of grid refinement from coarse (G1) to fine (G4) on Nu_a_ for Ra = 10^5^, φ = 0.045, and D = 0.1. The calculation becomes more precise as the grid captures more detailed physical phenomena, as evidenced by the increase in Nu_a_ as the grid is refined. Percentage and incremental increases are indicative of this. The minimal increase in Nu_a_’s from G3 to G4 indicates that the nearing of grid independence is reached, as further refinement does not substantially alter the obtained results. This is indicative of the potential for G3 to provide a mesh that is sufficient to achieve accurate and stable results without the unnecessary computational cost of further refinement, demonstrating a balance between computational time efficiency and accuracy.

**TABLE 2:** Grid sensitivity for Ra = 10^5^, φ = 0.045, and D = 0.1

	Nu_a_	Percentage increase	Incremental increase
G1: 95297	4.15447	—	—
G2: 241222	4.2812	3.05	—
G3: 720957	4.3835	5.51	2.39
G4: 3021847	4.4003	5.91	0.38

## 3 Results and discussion

This study aims to explore the enhancement of NC HT using HNF within a bi-truncated pyramid equipped with adiabatic cylinders. The purpose is to understand how varying Ra, φ , and D influence the thermal and fluid dynamics in complex geometries. The methodology involves a numerical simulation approach using the FEM to solve the governing equations of FF and HT. The study systematically varies the Ra from 10^3^ to 10^6^, φ from 0 to 0.045, and the cylinder diameter (D) from 0.05 to 0.24.

**FIGURE 3:**
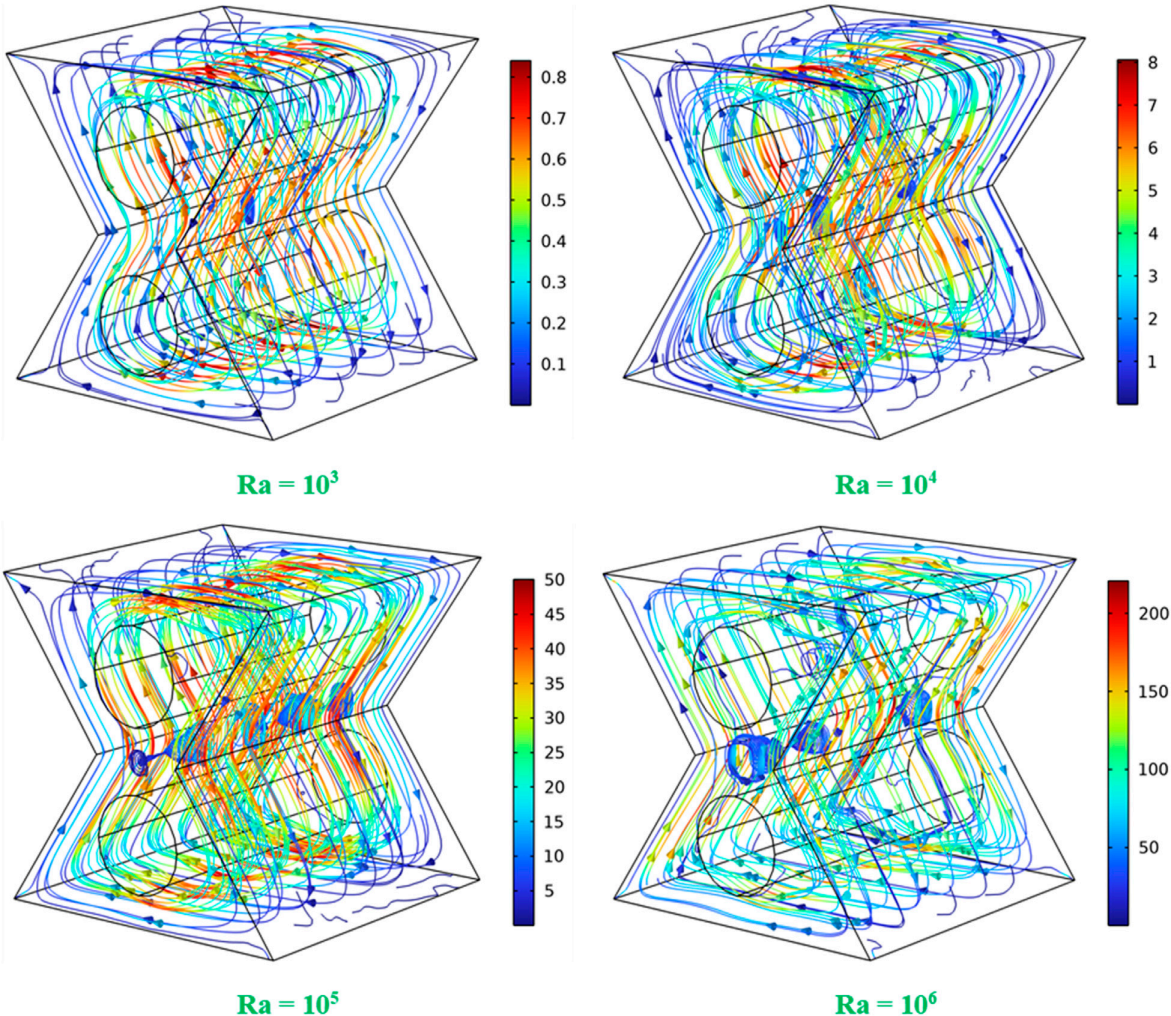
3D flow structures for φ = 0.045, D = 0.15 and various Ra values.

Figure 3 shows the flow patterns of the 3D convective flow in the hybrid nanofluid within the bi-truncated pyramid enclosure equipped with two internal adiabatic cylinders, for various Ra values (10^3^–10^6^). The left walls of the bi-truncated pyramid are hot, and the right walls are cold. This temperature differential is the driving force for the convective flow within the pyramid. As can be remarked from the figure’s legends, higher Ra numbers correspond to higher velocities within the fluid due to the increase of the buoyancy force with Ra. At Ra = 10^3^, the flow is more orderly with lower velocities. As Ra increases, higher velocities and more complex flow patterns, occur with the apparition of secondary flows between the cylinders. The most complex and intense flow structure occurs at Ra = 10^6^, where the highest velocities are reached, and additional secondary flow appear close to the cylinders. The figure illustrates the transition from orderly to complex convective flow as Ra increases from 10^3^ to 10^6^. Initially, at Ra = 10^3^, the HT is domination by conduction mode, and is characterized by smooth and coherent flow structures that indicate less intense convection due to the lower thermal driving force. As Ra increases, the impact of buoyancy forces overcomes the viscous damping and conduction, and the flow becomes more complex, signifying enhanced heat transfer. The presence of the two adiabatic cylinders, adds to the complexity by disrupting the flow, creating additional vortices and flow patterns around them.

**FIGURE 4:**
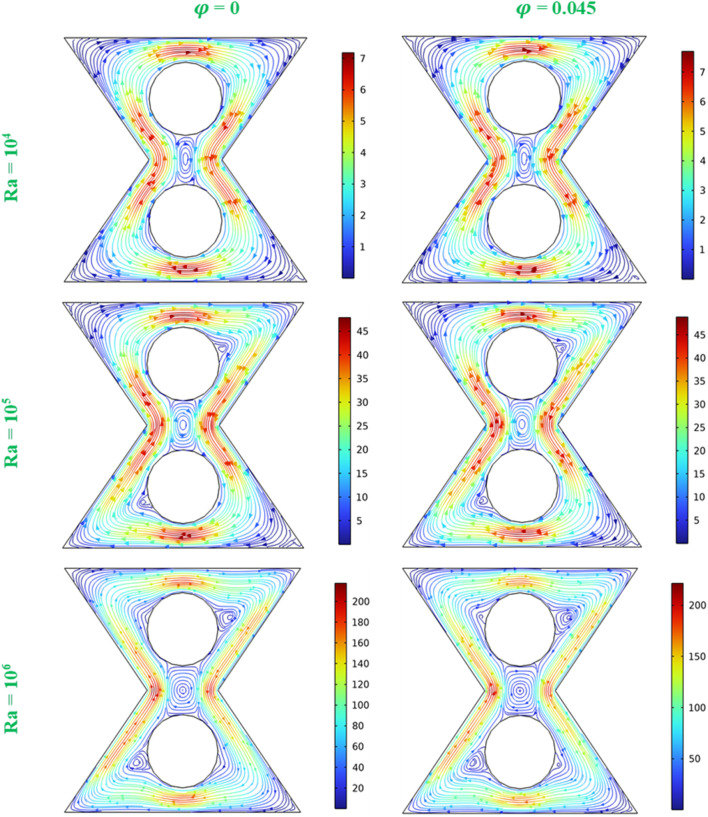
Streamlines at the central plan (z = 0.5), for D = 0.15, various Ra and φ values.

Figure 4 displays the streamlines at the central plane of the bi-truncated pyramid, comparing the flow patterns without nanoparticles ( φ = 0) and with nanoparticles ( φ = 0.045), at various Ra (10^4^, 10^5^, and 10^6^). Streamlines are presented for a better understanding of the flow of a hybrid nanofluid in the central cross-section of the pyramid. The presence of nanoparticles enhances the fluid’s thermal conductivity and influence the flow dynamics. At Ra = 10^4^, the flow is relatively moderate. Without nanoparticles ( φ = 0), the streamlines are orderly and symmetrical, showing a stable convective pattern. With nanoparticles ( φ = 0.045), the streamlines appear slightly more concentrated and exhibit stronger convection near the hot and cold walls, indicating that the nanoparticles are enhancing the convective motions. As Ra increases (10^5^), the effect of buoyancy becomes stronger, and we observe more pronounced convective vortexes. The presence of nanoparticles again amplifies the velocity, suggesting stronger convective flow and more efficient heat transfer. At the highest Ra (Ra = 10^6^), the flow pattern shows highly concentrated areas of streamlines, especially near the walls, indicating more intense convective motions. The addition of nanoparticles causes even tighter streamline patterns and larger gradients in the velocity field, suggesting an even stronger convective flow, which is indicative of enhanced heat transfer efficiency. The velocity fields for φ = 0.045 are more intense than that for φ = 0, this due to the modified thermal and flow properties of the fluid with nanoparticles. From this figure it can be concluded that, Without nanoparticles ( φ = 0), the flow is smooth, and the streamlines are well-ordered, suggesting a regular pattern of convection with moderate velocities. When nanoparticles are introduced ( φ = 0.045), the streamlines tighten and the flow velocities increase, particularly near the thermal boundaries, highlighting the nanoparticles’ role in enhancing thermal conductivity and the resultant convective HT. As the Rayleigh number escalates from 10^4^ to 10^6^, the streamlines in both cases become more compressed, indicating the strengthening in the buoyancy-driven flow. The nanoparticles amplify this effect, as the flow with φ = 0.045 consistently shows more intense streamlines and greater velocities. This effect is more pronounced at higher Ra, where the thermal driving force is greater, suggesting that nanoparticles could be instrumental in optimizing HT in engineering applications where controlling the flow intensity and heat distribution is crucial.

**FIGURE 5:**
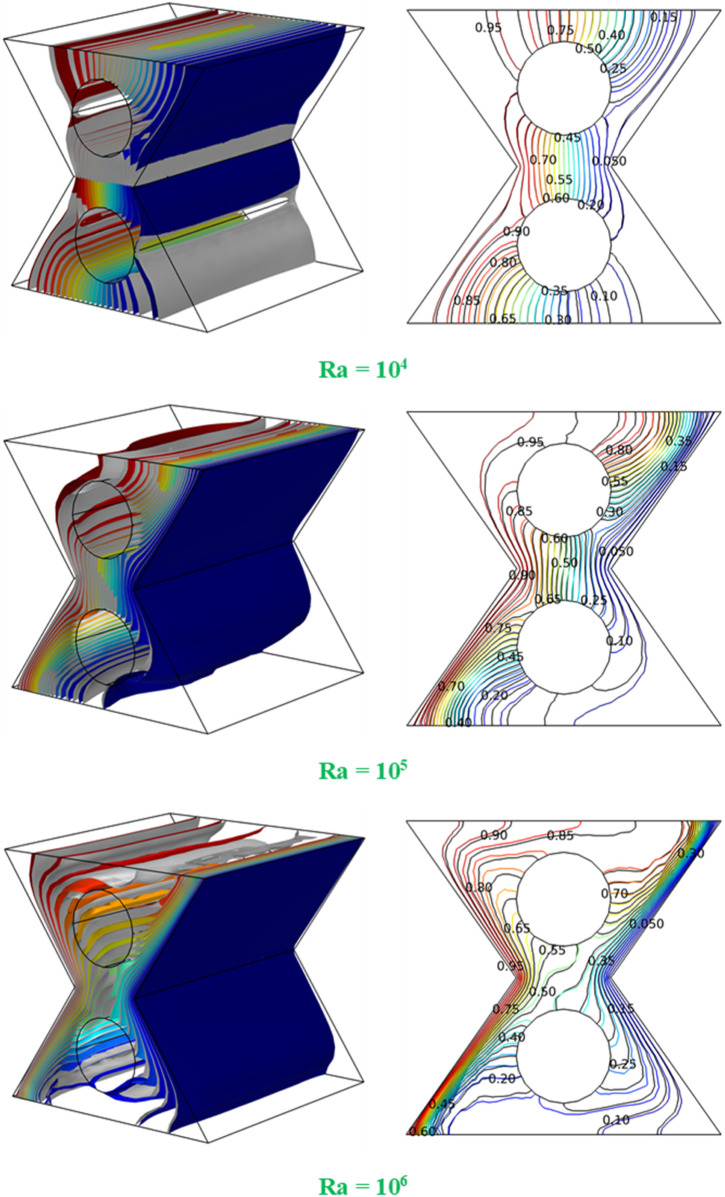
3D temperature field (left) and isotherm at the central plan for, D = 0.15 and various Ra; Colored ( φ = 0.045) and Gray ( φ = 0).

Figure 5 presents the 3D temperature field (left) and isotherms (right) at the central plane of the bi-truncated pyramid equipped with two internal adiabatic cylinders, for various Ra, and with a contrast between the pure fluid ( φ = 0) and the HNF ( φ = 0.045). At the low Ra (Ra = 10^4^), the temperature field is relatively undisturbed, indicating a weaker convective flow. The isotherms on the right-side show that with nanoparticles (colored iso-lines), the temperature gradient is more pronounced near the cylinders, suggesting that the nanoparticles enhance thermal mixing. At intermediate Ra (Ra = 10^5^), the temperature field becomes more varied, and the convective patterns are more evident, particularly in the presence of nanoparticles. The isotherms for φ = 0.045 are more tightly packed near the hot and cold boundaries, which points to a stronger temperature gradient due to the improved HT properties of the HNF. At the highest Ra (Ra = 10^6^), the temperature gradients are extremely sharp, and the influence of convection is dominant. For φ = 0.045, the isotherms depict a very concentrated thermal field near the hot surface, implying an intense heat transfer regime, likely resulting from the combined effects of increased thermal conductivity and convective enhancement provided by the nanoparticles. In addition, a vertical stratification of the isotherm appears and is more pronounced for the NF compared to the pure water. This figure put in evidence the critical role that nanoparticles play in manipulating the thermal characteristics of a fluid. The presence of nanoparticles tends to sharpen temperature gradients and enhance heat transfer, especially as the convective forces become stronger with higher Ra. The internal adiabatic cylinders serve to complicate the flow and temperature fields, acting as obstacles that induce local variations in heat transfer and fluid movement. From this figure, it can be concluded that at the lower Ra, the temperature fields and isotherms are relatively uniform, indicating moderate convective heat transfer. As Ra increases, the introduction of nanoparticles ( φ = 0.045) considerably alters the temperature distribution, as seen by the compressed isotherms, particularly around the hot and cold boundaries, signifying intensified thermal gradients and stronger convective currents. At the highest Ra, the impact of nanoparticles is the most pronounced, with the thermal field exhibiting tight gradients and complex patterns, indicative of a robust convection-driven heat transfer process. This thermal behavior suggests that nanoparticles effectively promote the heat transfer, which is a desired outcome in applications aiming for efficient thermal management and enhanced heat dissipation.

**FIGURE 6:**
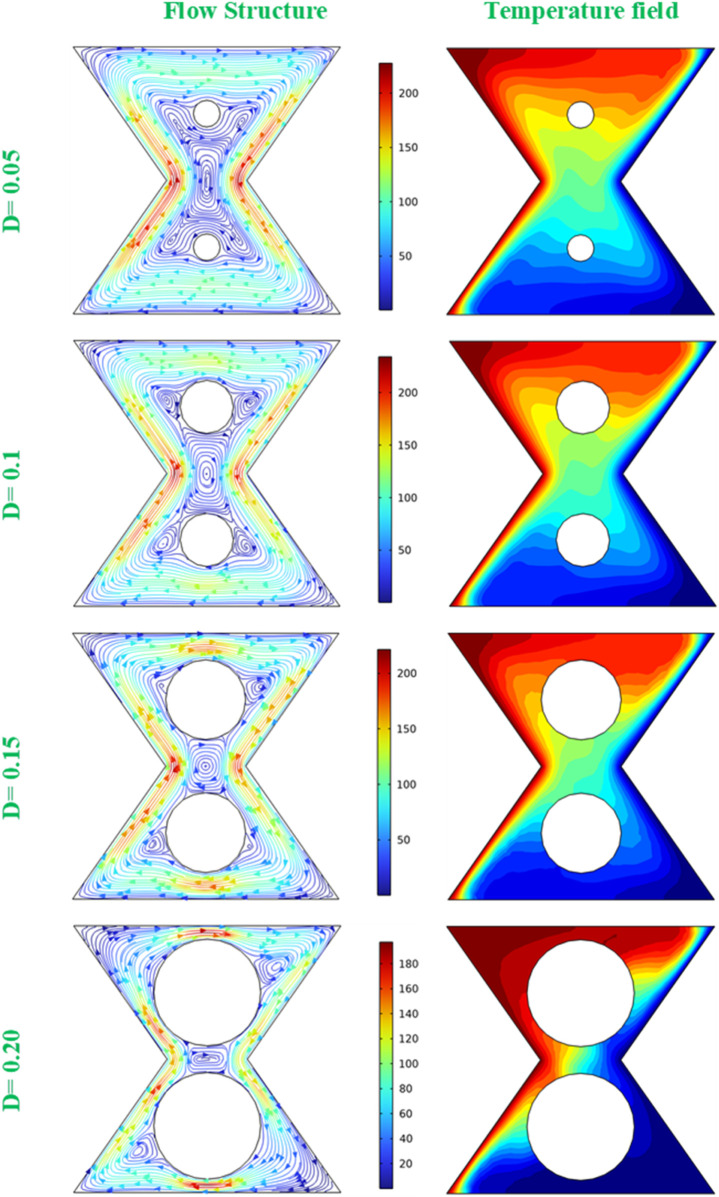
Flow structure (left) and temperature field (right) at the central plane for Ra = 10^6^, φ = 0.045 and various D values.

Figure 6 presents the flow structure and temperature field at the central plane of the bi-truncated pyramid at φ = 0.045, Ra = 10^6^ and various diameters (D) of the internal adiabatic cylinders. The streamlines represent the flow patterns within the central plane of the pyramid. As the diameter of the adiabatic cylinders increases from D = 0.05 to D = 0.24, the streamlines indicate changes in flow behavior around the cylinders. With smaller diameters, the flow can move around the cylinders more freely, which leads to a more uniform flow field. When the cylinder size is increased, the obstruction to the flow becomes more significant, which leads to a more disturbed flow patterns and to the apparition of secondary vortexes. The temperature distribution is presented to show how the heat is transferred from the hot to the cold walls of the pyramid. For small cylinder diameters (D = 0.05), a smooth transitions of the temperature gradients across the entire domain, occurs, this is an indication of the improvement of the efficient HT from the hot to the cold side. When the cylinder diameter is increased, the temperature field becomes more uneven, particularly around the cylinders. This is an indication that the larger cylinders imped the heat flow, creates cooler regions behind them, and potentially reduces the overall heat transfer efficiency. The figure gives an illustration of how the variation of the diameter of internal adiabatic cylinders affects both the convective flow patterns and the thermal fields. In fact, for the smallest cylinders (D = 0.05), the flow is less disrupted, and leads to a more homogeneous pattern, corresponding to the smoother temperature gradients, indicating that the HT is more efficient. As cylinder diameter increases, the flow encounters greater resistance, generating complex patterns that suggest enhanced fluid velocity, yet also revealing more pronounced thermal stratification with cooler regions behind the cylinders. This indicates a trade-off: larger cylinders augment fluid intensity but also create areas of thermal stagnation, potentially impacting the system’s overall HT efficacy.

**FIGURE 7:**
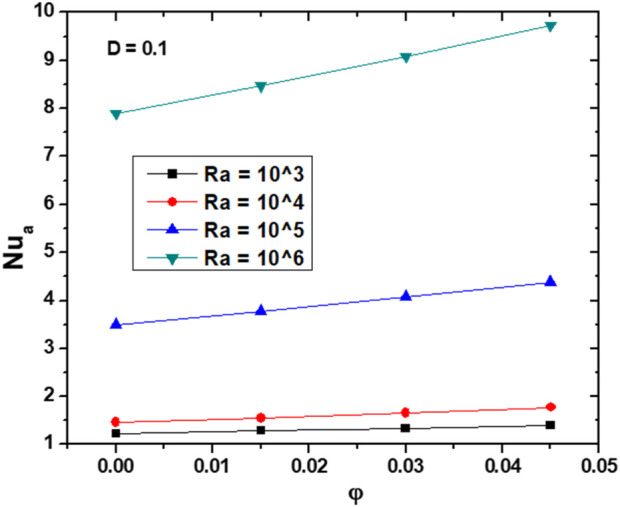
Effect of φ on Nu_a_ for D = 0.1 and various Ra values.

Figure 7illustrates the effect of φ on Nu_a_ at the left hot walls, for D = 0.1, across a range of Ra. When Ra increases, there is a consistent rise in the Nu_a_, indicating an enhancement in convective HT caused by the intensification of the buoyancy forces. This effect is furthermore intense for higher nanoparticles volume fractions; particularly at Ra = 10^6^; in fact, the slope of the curve is steeper, indicating better heat transfer improvement capabilities. At low Ra values, Nu_a_ slightly varies with varying φ , this is due to the dominance of the conductive HT regime where nanoparticles addition has minimal impact. This behavior changes at higher Ra values, where the introduction of NPs disrupts the thermal boundary layer more effectively, enhancing the fluid’s thermal conductivity and convective HT efficiency.

**FIGURE 8:**
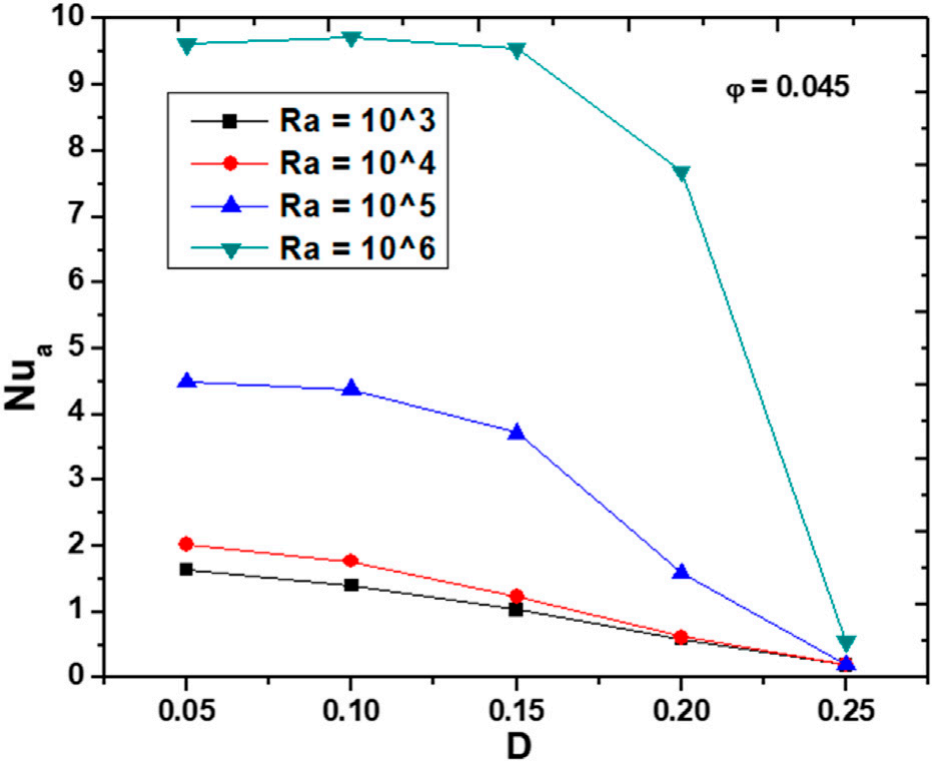
Effect of the obstacle diameter on Nu_a_ for φ = 0.1 and various Ra values.

Figure 8 illustrates the impact of increasing the diameter of internal adiabatic obstacles (D) on Nu_a_ at φ = 0.045, and different Ra values. As the diameter of the obstacles increases, there is a general decrease in Nu_a_ for all Ra values. This trend is more pronounced at higher Ra values. Specifically, at Ra = 10^6^, Nu_a_ exhibits a sharp decline as D increases, indicating that larger obstacles significantly reduce the fluid flow, thereby reducing heat transfer efficiency. In contrast, at lower Ra values, the decrease in Nu_a_ is less steep, thus it can be concluded that the influence of the adiabatic obstacles on the HT is less critical under weaker convection. This pattern highlights the nuanced relationship between obstacle size and convective heat transfer: larger obstacles disrupt the fluid path more extensively, creating regions of reduced fluid movement and increased thermal resistance. Conversely, smaller obstacles allow for more effective fluid navigation around them, maintaining better convective heat transfer.

### 3.1 Regression analysis

The inclusion of regression analysis provides a predictive tool for understanding the influence of key parameters, such as Rayleigh number, nanoparticle volume fraction, and cylinder diameter, on heat transfer performance. Practically, this regression model can be utilized by engineers and designers to optimize thermal systems without the need for extensive simulations. For instance, it can help determine the ideal combination of geometric and fluid properties to maximize heat transfer efficiency. The used polynomial regression extends linear regression by including polynomial terms in the model, which helps capture non-linear relationships between the independent and dependent variables. In our case, we fit a polynomial regression model to better represent the relationship between Nu_a_ and the governing parameters D, Ra, and ϕ. The resulting equation is as follows:

$$\begin{array}{ll} Nua= & 0.8971935-1.339456\times 10^{-9}\times D+4.106803\times 10^{-5}\times Ra\\ & +3.907659\times 10^{-7}\times \phi -8.272600\times 10^{-10}\times D^{2}\\ & +4.106803\times 10^{-5}\times D\times Ra+1.594561\times 10^{-7}\times D\times \phi \\ & -2.501650\times 10^{-14}\times {Ra}^{2}+1.601234\times 10^{-6}\times Ra\times \phi \\ & +2.001234\times 10^{-6}\times \phi^{2} \end{array}$$

With: R^2^ = 0.97.

The Mean Squared Error (MSE) value is 0.24 for the polynomial regression model indicating that, on average, the squared deviation between the predicted and actual values of Nu_a_ is minimal. An MSE of 0.24 indicates that the model accurately captures the relationship between Nu_a_ and the governing parameters.

This regression model elucidates the interplay between D, Ra, and φ in influencing the Nu_a_. The model reveals that while both the Ra and φ positively contribute to Nu_a_, indicating that enhanced thermal gradients and improved thermal properties of the fluid respectively facilitate heat transfer, the effect of the obstacle’s diameter is more complex. Specifically, a larger diameter initially boosts Nu_a_, by altering flow patterns to favor heat exchange, but the negative coefficients of its squared term and interactions suggest diminishing returns or even negative impacts at higher values, this due to flow disruption and increased resistance to convective flow.

**FIGURE 9:**
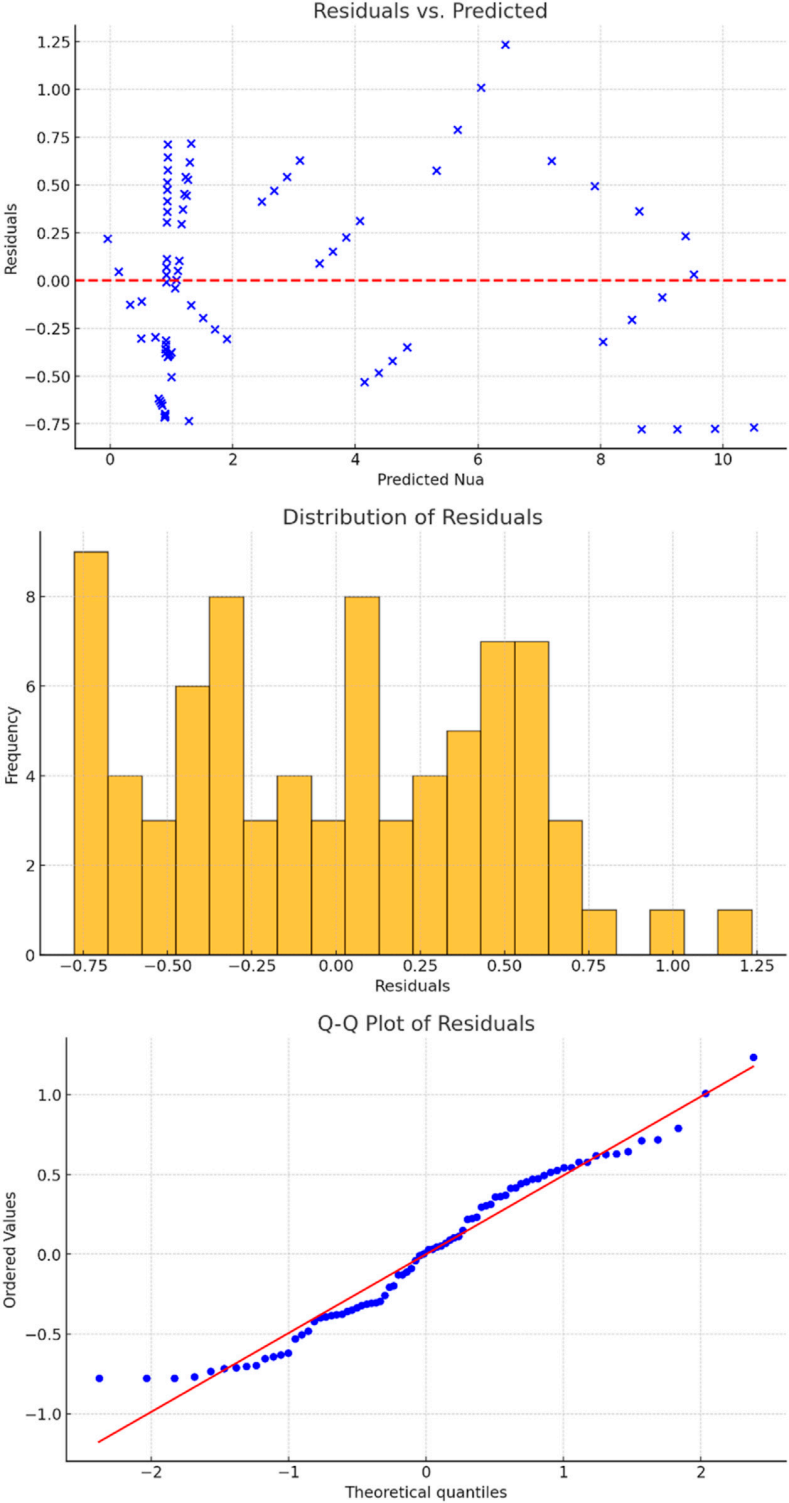
Residuals vs. Predicted, Distribution of Residuals and Q-Q plot for average Nusselt number.

As presented in Figure 9, the residual diagnostics give an idea on the performance of the regression model. The Residuals vs. Predicted plot shows a mostly random scatter of residuals around zero, which indicates that the model does not exhibit strong bias or systematic error. Thus, it can be concluded that the model captures the overall trend in the data effectively, though there may be minor non-linear effects that are not fully accounted for. The Distribution of Residuals (histogram) reveals that the residuals are symmetrically distributed around zero, closely resembling a normal distribution, which is a key assumption of linear regression models. However, there are slight deviations from perfect normality, as evidenced by the Q-Q Plot. While most points lie along the reference line, indicating that the residuals follow a near-normal distribution, there are small deviations at the tails. These deviations may indicate the presence of outliers or slight non-normality, but overall, the model performs well. In summary, the residual analysis suggests that the model fits the data well, with no significant violations of assumptions, though some refinement could be explored for extreme values.

## 4 Conclusion

The current study investigates the effectiveness of HNFs in enhancing natural convection HT within a bi-truncated pyramid. The simulations demonstrate that increasing Ra and φ significantly improve the HT efficiency, while the adiabatic cylinders size has an opposite effect by damping the flow and reducing convection. These findings highlight the potential of hybrid nanofluids to optimize thermal management systems, particularly in applications requiring precise control of heat transfer processes. Future research should explore the influence of different nanoparticle combinations and geometric configurations to further refine these enhancements and broaden their applicability in various engineering sectors. The main Results can be summarized as follows:

• The Nusselt number increased by up to 23% at Ra = 10^6^ and φ = 0.045.
• Optimal heat transfer efficiency was observed at a cylinder diameter of D = 0.15, which provided a balance between flow disturbance and thermal performance.
• Higher Rayleigh numbers contributed to more complex convective patterns, enhancing the overall HT efficiency.
• The presence of adiabatic cylinders created secondary vortices, further intensifying the FF and HT.
• The residual diagnostics of the regression model showed that the established expression of Nua fits well with the original data.

Future research will focus on optimizing heat transfer in 3D geometries by exploring the effects of varying cylinder shapes, positions, and materials, as well as incorporating advanced hybrid nanofluid formulations with enhanced thermophysical properties. Additionally, the integration of external influences such as magnetic fields, vibration effects, or transient thermal boundary conditions.

## Data Availability

The original contributions presented in the study are included in the article/supplementary material, further inquiries can be directed to the corresponding author.
